# Protective effect of *KCNH2 *single nucleotide polymorphism K897T in LQTS families and identification of novel *KCNQ1 *and *KCNH2 *mutations

**DOI:** 10.1186/1471-2350-9-87

**Published:** 2008-09-23

**Authors:** Xianqin Zhang, Shenghan Chen, Li Zhang, Mugen Liu, Sharon Redfearn, Randall M Bryant, Carlos Oberti, G Michael Vincent, Qing K Wang

**Affiliations:** 1 Key Laboratory of Molecular Biophysics of the Ministry of Education, College of Life Science and Technology and Center for Human Genome Research, Huazhong University of Science and Technology, Wuhan, Hubei 430074, PR China; 2 Department of Molecular Cardiology, Lerner Research Institute, Cleveland Clinic, and Department of Molecular Medicine, Cleveland Clinic Lerner College of Medicine of Case Western Reserve University, Cleveland, Ohio 44195, USA; 3 Department of Internal Medicine, LDS Hospital and University of Utah School of Medicine, Salt Lake City, Utah 84103, USA; 4 Department of Pediatric Cardiology, University of Florida Health Science Center, Jacksonville, FL, USA

## Abstract

**Background:**

*KCNQ1 *and *KCNH2 *are the two most common potassium channel genes causing long QT syndrome (LQTS), an inherited cardiac arrhythmia featured by QT prolongation and increased risks of developing *torsade de pointes *and sudden death. To investigate the disease expressivity, this study aimed to identify mutations and common variants that can modify LQTS phenotype.

**Methods:**

In this study, a cohort of 112 LQTS families were investigated. Among them two large LQTS families linkage analysis with markers spanning known LQTS genes was carried out to identify the specific gene for mutational analysis. All exons and exon-intron boundaries of *KCNH2 *and *KCNQ1 *were sequenced for mutational analysis.

**Results:**

LQTS-associated mutations were identified in eight of 112 families. Two novel mutations, L187P in *KCNQ1 *and 2020insAG in *KCNH2*, were identified. Furthermore, in another LQTS family we found that *KCNH2 *mutation A490T co-segregated with a common SNP K897T in *KCNH2*. *KCNH2 *SNP K897T was reported to exert a modifying effect on QTc, but it remains controversial whether it confers a risk or protective effect. Notably, we have found that SNP K897T interacts with mutation A490T in *cis *orientation. Seven carriers for A490T and the minor allele T of SNP K897T showed shorter QTc and fewer symptoms than carriers with A490T or A490P (*P *< 0.0001).

**Conclusion:**

Our family-based approach provides support that *KCNH2 *SNP K897T confers a protective effect on LQTS patients. Our study is the first to investigate the effect of SNP K897T on another *KCNH2 *mutation located in *cis *orientation. Together, our results expand the mutational and clinical spectrum of LQTS and provide insights into the factors that determine QT prolongation associated with increased risk of ventricular tachycardia and sudden death.

## Background

Long QT syndrome (LQTS) is an inheritable cardiac ion channelopathy characterized by prolonged QT intervals and characteristic T wave morphology changes on electrocardiograms (ECG). Patients with LQTS have increased risks of developing life-threatening polymorphic ventricular tachycardia/fibrillation (VT/VF) when subjected to physical or emotional stress, or upon exposure to QT prolonging drugs. VT/VF can result in cardiac events such as syncope, cardiac arrest and sudden death [1]. LQTS-related cardiac events often occur in the young, otherwise healthy individuals, and sudden death may present as the first symptom in many cases.

Multiple genes have been found to cause LQTS and these include *KCNQ1 *(LQT1), *KCNH2 *(LQT2), *SCN5A *(LQT3), *ANK2 *(LQT4), *KCNE1 *(LQT5), *KCNE2 *(LQT6), *KCNJ2 *(LQT7), *CACNA1C *(LQT8), *CAV3 *(LQT9) and *SCNB4 (LQT10)*. LQT4-10 are rare and there is a controversy as whether some of the subtypes are truly LQTS. LQT1 and LQT2 remain the mainstay as the most common LQTS genotypes. Previously, we reported a study of mutational analysis of *KCNQ1 *in 102 families and patients with a family history of lethal cardiac events, including LQTS [2]. The present study was carried out as a continued investigation in the same cohort of patients plus 10 new LQTS families/patients enrolled since the earlier report, and focused on mutational analysis of the *KCNQ1 *gene in the new families/patients and the LQT2 gene, *KCNH2 *in the whole cohort. Multiple mutations were identified. Most importantly, we provide the first family-based genetic evidence to show that two variants in *KCNH2*, mutation A490T and a common single nucleotide polymorphism (SNP) K897T, interact with each other in *cis *orientation to reduce the risk of LQTS (shorter QTc, less severe symptoms).

## Methods

### Study subjects

Patients with a clinical diagnosis of LQTS or idiopathic VT/VF and their first degree blood related family members were enrolled from medical clinics in North America. A total 154 families/patients were involved in this study. This study was approved by the Cleveland Clinic Foundation Institutional Review Board on Human Subject Research and informed consent was obtained from the study participants or their guardians.

### Clinical examinations

Diagnosis of LQTS was based on the QT interval and T wave morphology from 12-lead ECG and medical history of syncope, cardiac arrest and sudden death as described previously [3-7]. All the 12-lead ECG tracings available to the study were recorded at 25 mm/sec as standard in North America. QT interval was manually measured in leads II or V5 or the lead with longest QT interval and QTc was calculated using Bazett's formula for heart rate correction. A LQTS clinical diagnosis was considered if QTc ≥ 0.47 s in males or QTc ≥ 0.48 s in females [8]. Borderline QT prolongation (0.45–0.47 s) associated with symptoms were also considered as affected. Asymptomatic family members with QTc of ≤0.44 s and with a normal T wave morphology were phenotyped as non-affected. Other individuals were classified as having uncertain phenotype with regard to LQTS.

For the purpose of LQTS diagnosis, we used a stationary bike (ergometer) exercise protocol as described previously[9]. Superior to treadmill tests, ergometer tests eliminates the upper body movement to reduce ECG artifacts. An accurate QT measurement therefore can be achieved for exercise ECG recordings. 24-h Holter ECG monitoring was performed using Lifecard CF Digital Holter Recorder (Renolds Medical). LQTS phenotyping including ECG measurement was performed by G.M.V. and R.B. prior to the genetic testing.

For the bike test, we recorded standard 12-lead ECG in a paper speed of 25 mm/sec with voltage calibrated as 1 mV = 10 mm (or 10 mm/mV) for the baseline in supine and sitting positions, and exercise (3, 6, 9, 12, 15 minutes) and recovery (1, 3, 5 and 8 minutes) stages. QT interval was measured in the lead with the longest QT interval (mostly in lead II or V5) as described previously[9].

### Isolation of human genomic DNA

Each study participant donated 5–20 ml of blood samples, which were used for isolation of genomic DNA using the DNA Isolation Kit for Mammalian Blood (Roche Diagnostic Co.)

### Linkage analysis

For the two large families with LQTS, linkage analysis with markers spanning known LQTS genes was carried out to identify the specific gene for mutational analysis. Polymorphic microsatellite markers linked to LQTS genes were selected from the ABI PRISM Linkage Mapping Set-MD10 panel for linkage analysis, including *D11S4046 *for *KCNQ1*, *D7S798 *for *KCNH2*, *D3S1277 *for *SCN5A*, *D4S402 *for *ANKB *and *D21S266 *for *KCNE1 *and *KCNE2*. Markers were genotyped using an ABI 3100 Genetic Analyzer with the standard protocols provided by Applied Biosystems (Foster City, CA). Genotypes were analyzed using the GeneMapper 2 Software program (Applied Biosystems, Foster City, CA).

### Mutational and single-strand conformational polymorphism (SSCP) analyses

Mutational analysis was carried out using direct DNA sequence analysis. All exons and exon-intron boundaries of *KCNQ1 *and *KCNH2 *were amplified by polymerase chain reaction (PCR) using primers previously published [10-12]. PCR was performed in 10 μl of standard PCR buffer containing 1.5 mM MgCl_2_, 0.2 mM of each dNTP, 0.5 μM of each primer, 0.1 unit of Taq DNA polymerase, and 25 ng of DNA. The amplification program was one cycle of denaturation at 94°C for 5 min, 35 cycles of 94°C for 30 s, 62°C or other annealing temperatures for each specific pair of primers for 30 s, and 72°C for 1 min, and one 7 min extension step at 72°C. The PCR products were purified using the QIAquick Gel Extraction Kit (Qiagen Inc., Valencia, CA), and sequenced with both forward and reverse primers. DNA sequencing analysis was performed using the BigDye Terminator Cycle Sequencing v3.1 kit (Applied Biosystems, Foster City, CA).

SSCP analysis was used to determine whether a mutation co-segregated with the disease in the family and whether the mutation was present in normal controls. SSCP analysis was carried out as described previously [5,6].

### Statistical analysis

Statistical differences between groups were analyzed by Student's t tests and were considered significant when *P *< 0.01.

## Results

We investigated 112 families with a family history of lethal cardiac events, including LQTS, for mutations and common variants in *KCNQ1 *and *KCNH2*, the two most common potassium channel genes associated with LQTS. LQTS-associated mutations were identified in eight families. No mutation was detected in the rest of 104 families. We carried out mutational analysis in 112 families with a family history of lethal cardiac events, including LQTS. LQTS-associated mutations were identified in eight families. No mutation was detected in the rest of 104 families. The results are described below.

### Identification of two co-segregating variants, K897T and A490T of KCNH2 associated with LQTS in a mid-size family

**Figure 1:**
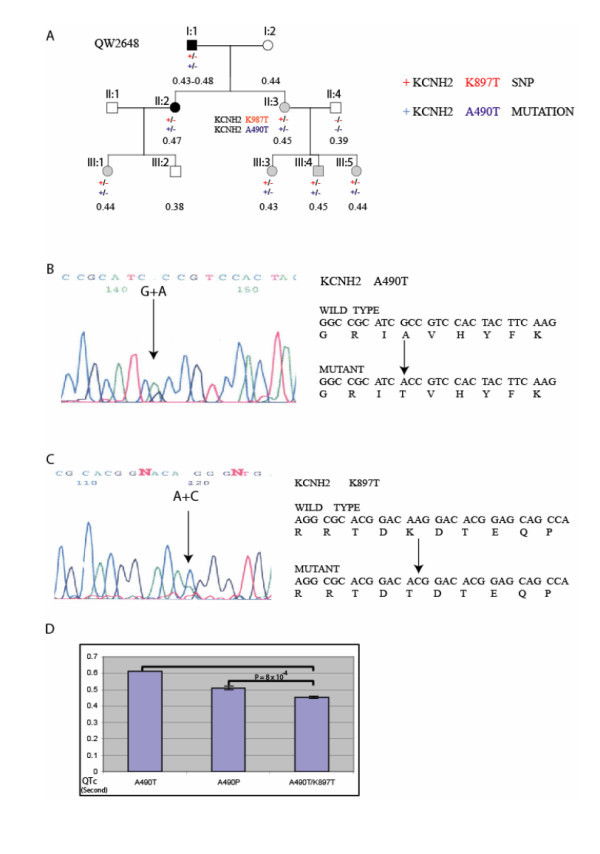
**Identification and co-segregation of *KCNH2 *mutation A490T and SNP K897T in family QW2648 and with LQTS.** (A). Pedigree structure of family QW2648. All the affected individuals carry double variants, K897T and A490T of *KCNH2 *on the same chromosome (in cis orientation). (B, C). DNA sequence analysis for the proband in family QW2648. A heterozygous G→A transition at nucleotide 1468 of *KCNH2*, which results in a substitution of alanine by threonine (A490T) (B). DNA sequence for *KCNH2 *SNP K897T is shown in (C). (D) Comparison of mean QTc for carriers with *KCNH2 *mutation A490T, A490P, and both A490T and SNP K897T.

Family QW2648 is a LQTS family with 11 family members (Fig. 1A). Two family members, I:1and II:2, had QTc of 0.47 s or higher (Fig. 1A). Linkage analysis for family QW2648 showed linkage to *D7S798 *near the *LQT2 *locus (Fig. 1A). Direct DNA sequence analysis of the DNA from the proband in the family identified two variants in *KCNH2*: a heterozygous G→A transition at nucleotide 1468, which results in a substitution of amino acid residue alanine by threonine (A490T) (Fig. 1B) and a heterozygous A→C transition at nucleotide 2690, which results in a substitution of amino acid residue lysine by threonine (K897T) (Fig. 1C). In the family, A490T co-segregated with K897T, suggesting that the K897T and A490T variants are in the cis orientation on the same chromosome (Fig. 1A). The normal family members did not carry either of the two variants.

The clinical characteristics of seven mutation carriers in family QW2648 are shown in Table 1. The maximum QTc ranged from 0.43 s to 0.48 s (0.46 ± 0.01) and the minimum QTc was from 0.41 s to 0.47 s (0.43 ± 0.01). Exercise ECG testing was performed for three carriers (III-3, III-4, and III-5), and showed that QTc was significantly prolonged compared to QTc before testing (*P *= 0.002). Bradycardia, defined as a resting heart rate of under 60 beats per minute, was seen in five of seven carriers with an average heart rate of 46 beats/min by resting ECG (Table 1). Holter monitoring in III:4 and III:5 showed 27 and 32 runs of bradycardia, respectively and the slowest rate was 34 beats/min. Syncopal episodes were reported in carriers I-1 (once while on a high protein diet), II-3 (several at the age of 11), and III-3 (once at the age of 17), but not in other carriers. There was no family history of cardiac arrest or of sudden cardiac death.

**Table 1:** Clinical characteristics of mutation carriers with *KCNH2 *A490T/K897T in family QW2648

-	Age (yr)	Average HR (beats/min)	Low HR (beats/min)	Bradycardia (runs/24 h × beats/min)	High QTc (s)	Low QTc (s)	QTc exercise (s)	Syncope (times)
I:1	72	67	N/A	N/A	0.48	0.43	N/A	1
II:2	35	66	N/A	N/A	0.47	0.47	N/A	0
II:3	40	61	43	N/A	0.47	0.42	N/A	multiple
III:1	17	56	53	N/A	0.45	0.44	N/A	0
III:3	17	46	44	N/A	0.43	0.43	0.49	1
III:4	15	55	44	27 × 34	0.45	0.42	0.48	0
III:5	11	61	47	32 × 34	0.44	0.41	0.50	0
Mean	-	58.86	46.20	-	0.456	0.431	0.49	0
SEM*	-	22.25	1.55	-	0.007	0.007	0.006	-

### Identification of a novel KCNQ1 mutation, L187P, in a large family

**Figure 2:**
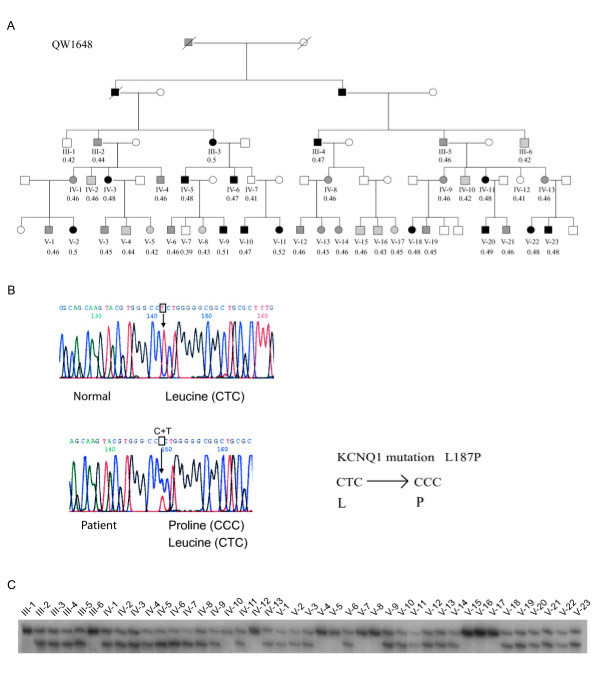
**Identification of a novel mutation, L187P, in *KCNQ1 *in LQTS family QW1648.** (A). A partial pedigree structure for the large Utah family is shown. The family had over 300 members. For ease of illustration only a portion of the pedigree is shown. Affected males and females are indicated by filled squares and circles, respectively. Normal individuals are shown as empty symbols. Individuals with uncertain LQTS diagnosis are shown with gray symbols. Deceased individuals are shown using slashes. The identification numbers for family members and QTc are shown below symbols. (B). DNA sequence analysis revealed a heterozygous T→C transition at nucleotide 560 of *KCNQ1*, which results in a substitution of amino acid residue leucine by a proline residue (L187P). The sequence for a normal family member is shown on the top and the sequence for an affected family member is shown below. (C). SSCP analysis showed co-segregation of the mutation L187P with the LQTS phenotype in the family. All phenotypically affected members had two bands, and the non-affected individuals had only one band.

We identified and characterized a 7-generation LQTS family with over 300 family members (QW1648, Fig. 2A). Five affected members in the family had a history of syncope. No aborted cardiac arrest was reported. One LQTS related sudden death had occurred before LQTS diagnosis.

DNA samples for forty-two family members from this large Utah family were available for genetic analysis. Linkage analysis with five markers for known LQTS genes revealed that the large Utah family was linked to *D11S4046 *at *LQT1 *on chromosome 11p15.5. The LOD score reached 6.11 at a recombination fraction of 0, which strongly suggests that the disease-causing gene in the family is *KCNQ1*. DNA sequence analysis of the DNA from the proband revealed a heterozygous T→C transition at nucleotide 560 of *KCNQ1*, which results in a substitution of amino acid residue leucine by proline (L187P) (Fig. 2B). The L187P mutation is located between domains S2 and S3 of KCNQ1.

To verify that the L187P mutation causes LQTS, SSCP analysis was carried out for the 42 family members with DNA samples. As shown in Fig. 1C, all affected members in the family carried the heterozygous mutation as demonstrated by the presence of two bands, whereas the normal family members showed one band, the wild type allele (Fig. 2C). The mutant SSCP band was not identified in 200 normal control individuals. These results suggest that the L187P mutation of *KCNQ1 *is a pathogenic mutation that causes LQTS in the family.

**Figure 3:**
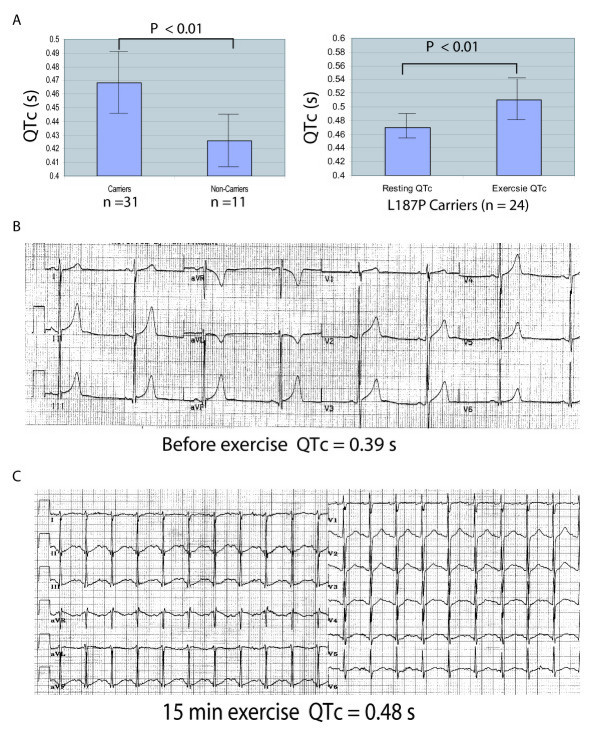
**(A) Reduced penetrance of LQTS phenotype and effect of exercise on QTc.** Mean QTc for carriers with mutation L187P and 11 non-carriers (left) and mean QTc for mutation carriers before (resting state) and during exercise (right). (B, C) Example of reduced penetrance of LQTS phenotype at baseline. Initial ECG (B) and ECG after 15-min exercise (C) are shown for a 29 year old, male, asymptomatic mutation L187P carrier.

Detailed clinical analysis revealed that the penetrance of the L187P mutation was not 100%. In the family, 31 of 42 family members genetically characterized here were identified as mutation carriers (age 33 ± 21 years, 13 females and 18 males) with mean QTc of 0.468 ± 0.022 sec and 11 of 42 members as non-carriers (age 30 ± 22 years, 4 females and 7 males) with mean QTc of 0.426 ± 0.019 sec (Fig. 3). Typical LQT1 T wave patterns were present in 84% of affected members. Only 16% were symptomatic. Among mutation carriers, 58% (18/31) had normal to borderline prolonged QTc (≤0.46 sec) on their initial ECGs, which overlapped with non-carriers (Fig. 3). A bicycle exercise test was performed for 24 mutation carriers and 4 non-carriers. Mean QTc was significantly prolonged during exercise in mutation carriers, whereas it remained in the normal range in non-carriers (Fig. 3). All mutation carriers with a normal or borderline prolonged QT interval reached QTc of 0.48 sec or greater during exercise tests.

### Identification of a novel 2-bp insertion mutation, 2020insAG in KCNH2

**Figure 4:**
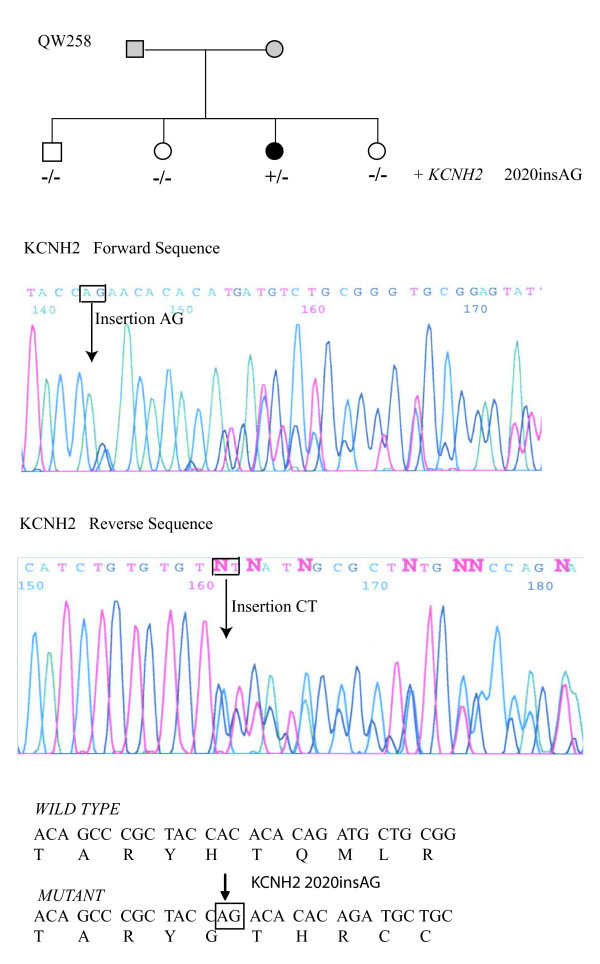
**Identification of a novel mutation in *KCNH2*, 2020insAG, in family QW258.** Top, pedigree structure; Middle, DNA sequence for the patient using the forward primer; Bottom, DNA sequence for the patient using the reverse primer.

One novel mutation, a 2-bp insertion 2020insAG, was identified in *KCNH2 *in family QW258 with LQTS (Fig. 4). The mutation was neither present in three normal family members nor 200 controls. The proband in the family was a 75-year-old female with a prolonged QTc of 0.596–0.687 s and left bundle branch block (QRS = 0.122 s). The patient developed a long run of polymorphic VT at the age of 75 years, which was cardioverted. She also developed two syncopal episodes at the age of 67 and 75 years, and a transient ischemic attack at the age of 63. Telemetry Holter ECG monitoring identified frequent multiform ventricular ectopic beats with occasional supraventricular ectopy, no bradycardia, and 173 runs of polymorphic VT. Echocardiography showed normal left and right ventricles, ejection fraction of 40% and abnormal left ventricular relaxation (stage I diastolic dysfunction). EEG, neurological and hearing tests, and troponin T and creatine kinase-MB levels (0.01 ng/ml and 4.8 ng/ml, respectively) were normal. Cardiac catheterization showed mild luminal irregularities without obstructing lesions, and mild global systolic left ventricular dysfunction consistent with hypertensive cardiomyopathy.

### Identification of five other mutations in KCNH2

DNA sequence analysis of the rest of families and patients identified five previously-known mutations in the *KCNH2 *gene. These mutations include A561T, D609N, A614V, N629S and R366X . Patients with mutations A561T, D609N, A614V, N629S all had a typical diagnosis of LQTS. Interestingly, the LQTS patient with mutation R366X also presented with bradycardia at the age of 30 years, *torsade de pointes*, recurrent syncopal episodes when awakening, startling and sitting, and first degree AV block.

## Discussion

In the present study, we carried out mutational analysis of *KCNQ1 *and *KCNH2*, the two most common potassium channel genes associated with LQTS in 112 families and patients with a family history of lethal cardiac events, including LQTS, Mutations were identified in 8 of 112 families. Interestingly, genotype-phenotype correlation studies in family QW2648 showed that two variants in *KCNH2*, mutation A490T and SNP K897T, interact with each other in *cis *to reduce the risk of LQTS (shorter QTc, less severe symptoms). As shown in Fig. 1, in LQTS family QW2648, two variants in the *KCNH2 *gene, one common SNP K897T and one mutation A490T, were identified. Because both variants co-segregated or in linkage disequilibrium in the family, they are located in the *cis *orientation on the same chromosome. A490T was previously reported as a *de novo *mutation in a Japanese female who showed markedly prolonged QTc of 0.61 s, bradycardia, *torsades de pointes *and recurrent syncope. Functional studies revealed that the mutant channel showed reduced current density by 39% compared to the wild type channel [13]. In a sharp contrast, none of the seven mutation carriers with A490T and K897T showed a QTc of > 0.48 s. The difference in QTc between A490T alone and combination of both A490T and K897T was notable (Fig. 3D). These results suggest that the T allele of SNP K897T confers a protective effect against QTc prolongation. On the other hand, SNP K897T did not appear to exert any effect on bradycardia because both A490T and A490T/K897T carriers exhibited clear evidence of the bradycardia phenotype. A mutation that changes A490 to P490 was recently reported in a large family with nine LQTS patients by Pellegrino et al [14]. The penetrance of mutation A490P was 100% in the family and the phenotype of LQTS in the family appears to be very severe. QTc ranged from 0.48 s to 0.57 s (mean QTc = 0.510 +/- 0.036), and five of the nine patients showed QTc of >0.50 s. Recurrent syncope, *torsade de pointes*, ventricular fibrillation, cardiac arrest, and family history of sudden death were documented in the family. The mean QTc between carriers with mutation A490P in comparison and those with both A490T and K897T was statistically significant (*P *= 0.0008, Fig. 3D). Our study is the first family-based genetic investigation to show that two variants in *KCNH2*, mutation A490T and SNP K897T, interact with each other in *cis *orientation and resulted in a mild LQTS phenotype.

Interestingly, no bradycardia was reported for mutations carriers with A490P, suggesting that the effect of mutation A490T on bradycardia may be mutation-specific. The molecular mechanism for mutation-specific development of bradycardia is unknown, but it is an interesting subject that warrants future investigation.

The modifying effect of *KCNH2 *SNP K897T has been studied previously, but inconsistency exists among different studies, mostly population-based studies. In this study, we investigated the effect of SNP K897T on QTc prolongation in multiple LQTS patients in a moderate-size family. This family-based approach has the advantage that the influence by environmental factors is more limited than a population-based approach. As described above, our results indicated that the rare T allele of SNP K897T has a protective effect against QTc prolongation (Fig. 3D). This is consistent with the results from a recent population-based study in 2,515 men and women involved in the Framingham Heart Study. In September, 2007, Newton-Cheh et al. showed that the common K allele of SNP K897T was associated with a 1.6 ms increase of QTc per allele copy [15], although the effect was very mild. Similarly, in 2005 in a population of 3,966 involved in the German KORA S4 study, Pfeufer et al. found that the rare T allele was associated with 1.9 ms and 3.5 ms decreases of QTc in heterozygotes and homozygotes, respectively [16]. In 2005, Gouas et al. studied a French population with 200 subjects with short QTc (100 men with QTc of 0.333–0.363 s and 100 women with QTc of 0.336–0.365 s) and 198 subjects with longer QTc (99 men with QTc of 0.394–0.433 s and 99 women with QTc of 0.400–0.432 s) [17]. The rare T allele was significantly more frequent in the group with short QTc than the group with long QTc (odds ratio = 0.64, *P *= 0.006), suggesting that the T allele was associated with shorter QTc. In 2003, Bezzina et al. found that TT homozygotes had shorter QTc than heterozygotes and KK homozygotes in a Caucasian population of 1,030 from Netherlands [18]. The association of SNP K897T with shorter QTc was not without controversy. In 2002, Pietila et al studied 267 women and 259 men from Finland and found that the rare T allele (KT + TT) was significantly associated with longer maximum QTc than the K allele (KK) in females only (0.441 s vs. 0.477 s, *P *= 0.005) [19]. In males, maximum QTc was longer in the KK group than the KT + TT group (0.465 s vs. 0.447 s), though the difference was not statistically significant (the small sample size may account for the finding of non-significance). In an Italian family Crotti et al. studied a small LQT2 family and reported a *KCNH2 *mutation (A1116V) and the T allele of SNP K897T on the non-mutant allele (i.e. in *trans *orientation) in one severe, symptomatic LQTS patient, whereas 3 relatives with A1116V alone were asymptomatic and showed mild QTc prolongation [20]. Thus, the T allele was associated with increased QTc in the Italian family, which is contrary to the results from our study. The *cis*-localization between the mutation A490T and SNP K897T in our family vs. *trans*-localization in the study by Crotti et al. may be one of the potential causes for the discrepancy. Overall, our results are more consistent with the finding that the minor allele T of SNP K897T plays a protective role on QTc lengthening.

There are some limitations for this study. First, the comparison of the mean QTc among carriers with A490T, A490P, and A490T/K897T was made in individuals of different geographical origins under different clinical settings. The accuracy of the analysis might be compromised as the ethnic and environmental influences have not been accounted for. Furthermore, the studied family was small. Second, no modifying variants in *SCN5A*, *KCNE1*, and *KCNE2 *were identified in the carriers with A490T/K897T in family QW2648, however, we cannot exclude the presence of variants in other ion channels that may influence QTc and other QTc-modifiers such as NOSAP1 variants [21]. Third, an *in vitro *functional study to examine the electrophysiological effects of the mutations or combinations of mutations may provide an insight into the molecular mechanism underlying the pathogenesis of LQTS.

In this study, we also identified two new mutations associated with LQTS, L187P in *KCNQ1 *and a *KCNH2 *mutation, the 2-bp insertion 2020insAG. The L187P mutation is located in the cytoplasmic loop between transmembrane domains S2 and S3 of the KCNQ1 channel. Two other mutations in the same area, G189R and R190Q, were loss-of-function mutations when expressed in *Xenopus *oocytes [22] Thus, the L187P mutation may also act by a loss-of-function mechanism. However, the mild phenotype of this large LQT1 family with 58% presented with a normal to borderline QT prolongation and only 16% of gene careers had a history of cardiac events, indicating that the functional outcome of L187P may be more complicated than a simple loss-of-function mechanism. The 2-bp insertion 2020insAG occurs at codon 674 and results in frameshift that leads to premature termination of translation. The mutant protein contains amino acid residues 1 to 673 of KCNH2 and addition of a short peptide, YQTHRCCGCGSSSASTRSPIPCASASRSTSSTPGPTPTASX. Alternatively, the frameshift creates an internal stop codon that leads to degradation of *KCNH2 *mutant mRNA by non-sense-mediated mRNA decay (NMD). A recent investigation that the NMD effect resulted in a mild phenotype in a large LQT2 family [23]. However, since >30% of *KCNH2 *mutations are nonsense mutations that may lead to NMD, a mutation that can trigger NMD effect may not necessarily lead to a mild LQTS phenotype. The investigation of modifier effects has opened a new chapter for us to better understand why some LQTS patients die suddenly and others do not. Genotype-phenotype correlation and further molecular electrophysiology investigation may help the risk stratification.

## Conclusion

In summary, our study identified two novel mutations causing LQTS, the L187P mutation in *KCNQ1 *and the 2020insAG mutation in *KCNH2*. Most interestingly, we showed that the rare T allele of SNP K897T conferred a protective effect on LQTS patients with *KCNH2 *mutation A490T located in *cis *orientation. Our study is the first to investigate the effect of SNP K897T on another *KCNH2 *mutation located in *cis *orientation. Together, our results expand the mutational and clinical spectrum of LQTS and provide insights into the factors that determine QT prolongation associated with increased risk of VT and sudden death.

## Competing interests

The authors declare that they have no competing interests.

## Authors' contributions

XZ and SC carried out genetic studies including linkage and DNA sequence analysis in the cohort of LQTS families and patients and controls, ML carried out linkage analysis and SSCP. LZ, SR, RB, CO, GMV performed the clinical characterization of the patients and family pedigree analysis. QKW supervised the study, obtained the funding, and critically revised and approved the manuscript. All the authors read and approved the final manuscript.

## Pre-publication history

The pre-publication history for this paper can be accessed here:


